# New Appliances and Things Medical

**Published:** 1902-03-08

**Authors:** 


### NEW APPLIANCES AND THINGS MEDICAL.

**[We shall be glad to receive, at our Office, 28 & 29 Southampton Street, Strand, London, W.C., from the manufacturers, specimens of all new preparations and appliances which may be brought out from time to time.]**

### BRAND'S VIRVIS AND FEVER FOOD.

(BRAND & CO., LIMITED, 11 LITTLE STANHOPE STREET, MAYFAIR, LONDON, W.

BRAND'S Virvis is a concentrated food which is particularly prepared for the nourishment of delicate and invalid children. It has an agreeable taste, something like honey, and is practically of the same consistency. Combining the blood-forming properties of red marrow with the nutritive qualities of eggs and meat, and possessing at the same time the digestive properties of a malt extract, this preparation might be safely employed as an exclusive food during temporary emergencies or acute illnesses. For the wasting conditions of infancy, such as atrophy and marasmus, it is particularly indicated. Brand Fever Food is another concentrated food with stimulating and tonic qualities. Combining the nutritive properties of eggs, butter, and essence of meat, it is clean tasting, and well suited to the capricious palate of the invalid.

### AQUARIUS WATER SOFTENER.

(LONDON AND GENERAL WATER PURIFYING CO., LTD., 157 STRAND, LONDON, W.)

THIS new water softener is a specially-prepared powder for providing hard water with the agreeable washing qualities of rain water. The powder is sold in air-tight tins at 2d., 4d., and 6d.; the contents of the latter are sufficient to soften 50 gallons of hard water. For toilet use the method of employing this powder is as follows: – Sprinkle a teaspoonful of the powder over the surface of the water in the ewer overnight. In the morning the water will be ready for use, clear and beautifully soft. A simple method, such as the one described and at such a very small cost, is well calculated to commend itself to all those who value their complexions and appreciate economy in soap.

### TAPPS' PATENT FUMIGATOR.

(AGENTS: S. MAW, SON AND SONS, 7–12 ALDERSGATE STREET, LONDON, E.C.)

THIS new fumigator is a simple, strong, and effective apparatus for disinfecting and deodorising rooms, buildings, operating theatres, etc. Briefly described, the principle on which this apparatus works is as follows:—A suitable fluid, such as formalin, carbolic acid, or eucalyptus oil, is contained in a double coned-shaped cistern, from which, by a special mechanical arrangement, the fluid flows drop by drop at any required rate on to a metal plate or receiver below, which is kept continuously hot by a small spirit lamp placed below. This central metal pan contains a shallow layer of ordinary silver-sand which retains the heat and facilitates the evaporation of the disinfecting or deodorising fluid. The lamp will burn for about 24 hours, while neither it nor the apparatus require any adjustment after once they have been set going. So simple and efficacious an arrangement for the automatic disinfection of rooms will be much appreciated by the medical and nursing profession.

